# The Relationship between Removable Dental Prostheses and Brain Activity in Elderly Individuals: Systematic Review

**DOI:** 10.5041/RMMJ.10538

**Published:** 2025-01-30

**Authors:** Arpit Sikri, Jyotsana Sikri, Rinshul Saroch, Charanjeet Singh Gill, Rimple Gupta, Chetan Pathak

**Affiliations:** 1 Department of Prosthodontics, Crown & Bridge, and Oral Implantology, Bhojia Dental College & Hospital, Baddi, Himachal Pradesh, India; 2 Department of Conservative Dentistry & Endodontics, Bhojia Dental College & Hospital, Baddi, Himachal Pradesh, India; 3 Department of Conservative Dentistry & Endodontics, Himachal Institute of Dental Sciences, Paonta Sahib, Himachal Pradesh, India; 4 Department of Conservative Dentistry & Endodontics, Guru Nanak Dev Dental College & Research Institute, Sunam, Punjab, India; 5 Department of Prosthodontics, Crown & Bridge, and Oral Implantology, Sudha Rustagi College of Dental Sciences and Research, Faridabad, Haryana, India

**Keywords:** Brain function, cognitive impairment, denture, oral rehabilitation, physical activity, prosthodontics, removable prostheses, tooth loss

## Abstract

**Background:**

There is an increasing body of literature associating edentulism with cognitive impairment. The aim of this systematic review was to summarize the available data, emphasizing the role of removable dental prostheses in preventing cognitive deterioration and promoting brain health in elderly individuals.

**Aim:**

This systematic review investigates the relationship between the use of removable dental prostheses and physiological or adaptive changes at the cerebral level in partially and completely edentulous patients.

**Methods:**

A systematic review was conducted following PRISMA guidelines, with an initial search across PubMed, Scopus, and Web of Science databases. Studies published up to June 2023 in English were considered. A risk of bias assessment was performed for included studies.

**Results:**

Of the 86 studies initially screened, 13 met the inclusion criteria. Findings indicate a positive association between the use of removable dental prostheses and improved cognitive function, with potential therapeutic implications for managing cognitive decline.

**Conclusion:**

Removable dental prostheses play a crucial role in enhancing neurological health and preventing cognitive decline, making them an important consideration in the management of neurodegenerative diseases.

## INTRODUCTION

Presently, life expectancy has shown remarkable improvement, thanks to advances in healthcare compared to previous eras. Nevertheless, this progress has brought about a rise in age-related ailments.^1^ Dentistry, too, is impacted by these developments, as tooth loss among the elderly is now a prevalent issue that hampers essential functions of the stomatognathic system, such as speech, swallowing, and chewing.^2^ Moreover, tooth loss has been identified as a potential risk factor for non-communicable diseases like diabetes, heart diseases, and Alzheimer’s.^3^ As a result, studies have demonstrated that oral health in older adults is a predictor of their overall quality of life, linked to the concept of healthy, successful, and active aging. Addressing these trends comprehensively requires consideration not only of the health aspect but also of personal well-being, functionality, and supportive socio-familial environments that enable them to continue their personal development.^4^

Similar to other healthcare fields, dentistry carries a responsibility to serve society.^5^ Embracing new preventive techniques, innovative restorative materials, and a shift towards minimally invasive procedures has become crucial.^6^ Despite these advancements, the utilization of dental services remains intricate, influenced by factors like the availability, acceptability, and accessibility of dental care.^7^ Consequently, new regulations for prosthetic treatments have emerged, emphasizing the importance of having dental surgeons capable of catering to the diverse demands of patients across various age groups.^8^

Currently, a common treatment for tooth loss is the use of removable dental prostheses.^9^ This treatment can lead to various biological and functional changes, especially in mastication, and several studies have found that it causes noticeable alterations in brain activity.^10^–^12^ Considering the significance of this topic for the health and quality of life of older individuals, it becomes imperative to analyze previously conducted research on this subject.

In the existing literature, no comprehensive review has been conducted on removable dental prostheses and alterations in brain activity, highlighting the need to assess the relevant information. The primary objective of this systematic review study was to explore the scientific literature concerning the association between removable dental prostheses and brain activity.

## METHODOLOGY

This systematic review sought to investigate whether there is a correlation between the utilization of removable dental prostheses and any physiological or adaptive alterations in cerebral activity among edentulous patients. Hence, relevant data were sought based on the following research question: “What is the comparative impact on cerebral activity effectiveness between edentulous patients who use removable dental prostheses and those who do not use any removable dental prostheses?”

### Protocol and Registration

This systematic review was designed and officially registered under the PROSPERO Registration number CRD42023450119.

### Search Strategy

The literature search utilized PubMed, Scopus, and Web of Science. A number of specific terms were included in the multiple search queries, including “dental prostheses and cognitive functioning,” “dental prostheses and brain function,” “tooth loss and cognitive loss,” “mastication and prefrontal activity,” and “prostheses and mental state.” Additionally, a manual search of the sources cited in the selected articles was done.

### Eligibility Criteria and Selection of Studies

The literature search was structured using the Population, Intervention, Comparison, and Outcome (PICO) strategy^13^ (Table 1). The Preferred Reporting Items for Systematic Reviews and Meta-Analyses (PRISMA) guidelines were also used, to ensure a transparent and comprehensive reporting process.^14^ The PRISMA checklist was referred to for guidance throughout development of the review methodology.

**Table 1:** PICO Strategy Used to Design the Research.

Parameter	Explanation
**P (Population)**	Elderly population, including both male and female individuals. These human subjects were of advanced age, commonly referred to as old-aged adults.
**I (Intervention)**	The utilization of removable dental prostheses in the elderly population aims to replace missing teeth, improving the cerebral activity.
**C (Comparison)**	Individuals receiving no prosthetic replacement for missing teeth and individuals with prosthetic replacements.
**O (Outcome)**	Effect of removable dental prostheses on enhancing the cerebral activity of elderly individuals.

During the document selection process, the following inclusion criteria were considered, with a specific focus on removable dental prostheses:

1. Language restrictions: publications in the English language only were considered, ensuring comprehension and accessibility.
2. Publication date: documents published through June 30, 2023 were included so the review would reflect the most recent research trends and findings based on well-established evidence and the latest available literature. This cutoff ensured incorporation of cutting-edge studies while removing potential biases from later publications that might not have undergone peer review.
3. Study type: eligible publications included scientific articles such as case studies, clinical studies, and control studies, as well as systematic reviews, for a more robust understanding of the topic.
4. Access: full-text articles and abstracts had to be accessible to enable comprehensive evaluation of their content.
5. Target population: studies focused on edentulous patients wearing removable dental prostheses ensured alignment with the target population.
6. Cognitive state: only studies involving patients without any cognitive impairment were included, to avoid confounding factors.
7. Outcome measures: included studies measured cerebral activity in patients undergoing rehabilitative treatment, specifically before and after rehabilitation, to assess cognitive impact.

Studies were excluded from consideration if they were not in English, were *in vitro* studies, or were considered to be non-scientific literature, such as essays, opinions, editorials, or perspective pieces.

Employing the above inclusion and exclusion criteria helped the researchers select scientifically robust studies directly related to the impact of removable dental prostheses on cerebral activity in edentulous patients.

### Data Extraction and Quality Assessment

The data extraction process employed a double-check mechanism and independent review, thereby minimizing potential biases or data extraction errors. This approach ensured that the included studies aligned with the predefined criteria and that the extracted data were accurate and reliable.

### Risk of Bias

Most of studies reviewed exhibited either an unclear or low risk of bias for random sequence generation, allocation concealment, performance, and detection bias, primarily due to incomplete or unclear protocols. Hence, there was uncertainty regarding the randomization process, participant allocation to study groups, how interventions were performed, and outcome assessments.

For this systematic review, the risk of bias in the included studies was assessed using the Cochrane Risk of Bias Tool, a standardized tool for evaluating potential biases in randomized controlled trials.^15^ This tool evaluates seven specific domains of bias:

1. Random sequence generation (selection bias)
2. Allocation concealment (selection bias)
3. Blinding of participants and personnel (performance bias)
4. Blinding of outcome assessment (detection bias)
5. Incomplete outcome data (attrition bias)
6. Selective reporting (reporting bias)
7. Other biases (e.g. baseline imbalance, deviations from intended intervention)

For each domain, the risk of bias was categorized as “low,” “moderate,” or “high,” based on the information provided in the studies.

### Review Process

To ensure objectivity and reliability, two independent reviewers assessed the risk of bias for each included study. They worked independently, with their evaluations blinded to the other’s assessments to minimize potential influence or bias. Any disagreements between the reviewers were resolved through discussion and, if needed, consultation with a third reviewer to reach a consensus.

### Documentation and Transparency

The criteria for evaluating each bias domain were documented in advance, following the Cochrane Handbook guidelines.^16^ A detailed table summarizing the risk of bias assessments for all included studies was subsequently compiled for incorporation in this manuscript. This table outlines the judgments for each domain of bias, along with justifications based on the available study data.

By employing this robust and transparent process, we ensured a comprehensive evaluation of the risk of bias, thereby maintaining the integrity of the study findings.

Each included study was evaluated for risk of bias based on common categories such as selection bias, performance bias, detection bias, attrition bias, reporting bias, and any other study-specific risks. Since we expected a mix of observational and interventional studies (systematic reviews, cohort studies, pilot studies), we adapted the appropriate criteria for each study, aided by bias-specific queries:

1. Selection bias: Was the study population representative of the target population? Was randomization used if applicable?
2. Performance bias: Were interventions or exposures applied consistently across the study groups?
3. Detection bias: Were outcome assessments conducted in a blinded manner, especially for subjective measures (e.g. cognitive function)?
4. Attrition bias: Did any participants drop out, and how was this addressed in the analysis?
5. Reporting bias: Were all outcomes planned and reported in the final study, or were selective outcomes published?

## RESULTS

**Figure 1:**
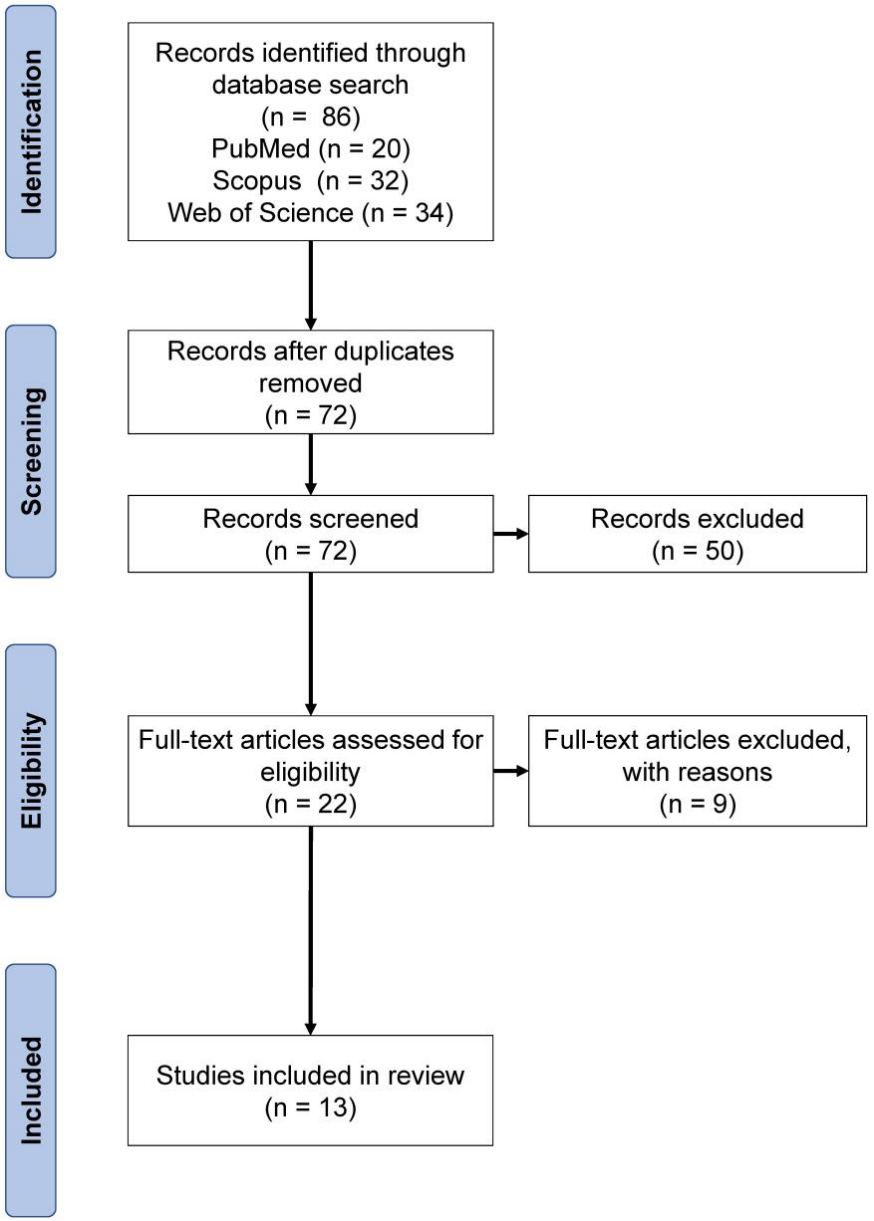
Selection of Articles According to PRISMA Protocol.

The article selection process used the PRISMA protocols (Figure 1).^14^ Initially, 86 documents matched the search criteria. After removing duplicates, 72 articles remained for abstract evaluation.

A total of 50 studies that did not involve elderly individuals were excluded, leaving 22 full-text publications. An additional 9 studies were excluded since they either focused on different populations or utilized fixed dental prostheses. This rigorous selection left 13 articles that met the inclusion criteria and were selected for further analysis. These studies were instrumental in addressing the research question and exploring the relationship between removable dental prostheses and cerebral changes in edentulous patients.

After data extraction, the findings from the included studies were organized and are presented based on various outcomes of interest (Table 2); the risk of bias for the studies is presented in Table 3. The key findings of the 13 studies are summarized below.

**Table 2:** Extraction of Data from the Included Studies.

Author (Year)^ref^	Objective	Results and Conclusions
Ko et al. (2022)^17^	To investigate the association between changes in masticatory function and cognitive impairment by analyzing longitudinal data of older Korean patients	Dementia group: T-FTU significantly decreased from T1 to T2 (9.81±2.78 to 9.11±3.16, respectively, *P*=0.008). Control Group: no significant change observed. Mean observation period of 9 years: significantly more teeth extracted and neglected, needing prosthetic restoration in the dementia group vs controls. Regression analysis: number of missing/neglected teeth (OR=1.195, 95% CI=1.025–1.393, *P*=0.023); previous alcohol consumption (OR=4.445, 95% CI=1.831–1.795, *P*=0.001) significant risk factors of dementia. Possible causative relationship between missing or neglected dentition and dementia onset.

Costa et al. (2022)^18^	To establish the relationship between oral prosthetic rehabilitation and the regional increase in brain activity	Two reviewers searched PubMed/MEDLINE, Embase, Cochrane Library, and https://clinicaltrials.gov databases up to June 2021. A total of 8 articles were included in the review, all of which identified a regional increase in blood flow and regional cerebral activity during dental prosthesis use. A positive association was found between different types of prosthetic rehabilitation and brain function. Prostheses may preserve and restore neurological health.

Ahmed et al. (2021)^19^	To assess the influence of dental prostheses on cognitive functioning in elderly population	19 studies met the inclusion criteria; 15 were selected, with 4 excluded from review with reason. The authors concluded that dental prostheses play a significant role in preventing cognitive impairment and act as a protective factor in enhancing cognitive function in patients with dementia-related and neurodegenerative diseases.

Moussa (2020)^20^	To evaluate the influence of restoring lost posterior occlusal contacts with removable partial dentures on brain activity and cognitive function in controlled type 2 diabetic patients	EEG assessment demonstrated an increase in mean value after 1 month of wearing removable partial dentures. Cognitive scores for MMSE displayed an increase in total mean value after 1 month; outcomes were statistically significant (*P*<0.05). Restoration of lost posterior occlusal contact in controlled type 2 diabetic patients with removable partial dentures improved brain function and cognitive status.

Tan et al. (2020)^21^	To investigate (a) changes in masticatory performance with POR, (b) the association between POR and neurocognitive function using fMRI, and (c) outcomes of OHRQoL.	Four edentulous patients (mean age: 73±1.4 years) received new complete RDPs which were later changed to mandibular RDPs with two retained implants (IR-RDPs). Improvements in masticatory performance and OHRQoL were observed from complete RDPs to IR-RDPs. Prosthetic adaptation was associated with neurocognitive changes, with activity levels approaching or exceeding those seen before insertion, over a 6-week period. These pilot data suggest both behavioral and neural associations between POR and cognition.
Chuhuaicura et al. (2019)^22^	The relationship between mastication as a protective factor of cognitive decline and the effect on brain blood flow in adults	Analysis and qualitative analysis of 9 clinical descriptive studies suggested that the most activated brain areas during mastication are the frontotemporal cortex, the caudate nucleus, and the thalamus. These findings revealed a positive correlation between chewing intensity and perfusion of the principal trigeminal nucleus. The study concluded that masticatory function may act as a protective factor in patients with cognitive impairment and neurodegenerative diseases. Several mechanisms, including the increase of cerebral blood flow, are suggested to be involved in this beneficial effect.

Fukutake et al. (2018)^23^	To determine the impact of cognitive function on perception in older adults living independently	This study comprised 987 participants (466 men, 521 women), aged 69–71 years. The number of teeth, use of removable dentures, and cognitive function, respectively, were significantly related to the stereognostic score. Cognitive impairment, even in the preclinical stage, was associated with reduced oral perception after controlling for gender, number of teeth, and use of dentures in older adults living independently.

Banu et al. (2016)^24^	To assess brain activity and cognitive function in edentulous patients under two conditions: edentulous, with conventional dentures, and edentulous mandibular IODs, considering mastication effects before and after treatment	The study included 10 patients aged between 55 and 65 years. The amplitude, power of alpha waves, and cognitive scores gradually increased, with the highest mid-range values observed for the IOD group. Findings highlighted the significance of two mandibular IODs in improving an individual’s mental state, primarily due to functional improvement with prostheses when loaded with implants rather than solely the presence of an implant without any function.

Matsuda et al. (2014)^25^	To identify how changes in the VDO affect sensory perception and brain activity in complete denture users, using EEG	The study included 21 patients (average age, 77 years). No significant differences were found in neuronal activity values before and after chewing gum with either of the dentures (*P*>0.05). However, a significant decrease in occlusal force was observed between the original denture and the denture with a −3 mm VDO (*P*<0.05). Both psychological state and occlusal force were influenced by immediate changes in the VDO of the complete denture.

Luraschi et al. (2013)^26^	To investigate cortical brain changes using fMRI following complete denture renewal, and to examine how these changes relate to prosthodontic treatment adaptability, as measured by chewing efficiency and maximum bite force	Cohort: 10 complete denture patients (mean age ± standard deviation: 70.3±9.1 years). Changes in brain activity occurred during adaptation to dental prostheses replacement and appeared to recover activity levels seen before insertion during motor tasks involving dental occlusion 3 months following insertion.
Ohkubo et al. (2013)^27^	To analyze the effect of occlusion on overall body health with a specific focus on brain function	The sensorimotor cortex was found to be affected by the placement of occlusal interference devices, splints, and implant prostheses. Brain activity can change depending on the force of movements in the oral and maxillofacial area. Therefore, chewing and other movements stimulate activity in the cerebral cortex, which may be helpful in preventing the degradation of brain function.
Kimoto et al. (2011)^28^	To investigate changes in regional brain activity during gum chewing when edentulous subjects transitioned from complete mandibular dentures to removable IODs	Results revealed that IOD treatment significantly suppressed brain activity induced by chewing in the prefrontal cortex. Induced brain activities in the primary sensorimotor cortex and cerebellum tended to decrease with IOD, although not statistically significant. No significant changes observed in brain activity in the supplementary motor area, thalamus, and insula between gum chewing with CD vs IOD. Statistical parametric mapping analysis demonstrated a significant decrease in neuronal activity in the frontal pole during gum chewing with IOD compared to CD (*P*<0.05), specifically within the prefrontal cortex. Gum chewing in elderly edentulous patients may result in differential neuronal activity in the frontal pole within the prefrontal cortex between the two prosthodontic treatments: mandibular CDs and IODs.

Hosoi (2011)^29^	The relationship between brain function activity, measured by EEG, and dental prosthesis treatment in elderly patients with complete and partially edentulous conditions	Treatment with complete dentures improved function and enhanced brain function activation. Patients evaluated according to Eichner classification: Use of partial dentures increased brain function activation after chewing. These results indicate that the occlusal contact area and occlusal force influence brain function activation.

CD(s), complete denture(s); CI, confidence interval; EEG, electroencephalogram; fMRI, functional magnetic resonance imaging; IOD(s), implant-supported overdenture(s); IR-RDPs, implant-retained removable dental prostheses; MMSE, Mini-Mental State Examination; OHQoL, oral health-related quality of life; OR, odds ratio; POR, progressive oral rehabilitation; RDPs, removable dental prostheses; T-FTU, total functional tooth unit; VDO, vertical dimension of occlusion.

**Table 3:** Risk of Bias Assessment.

Author (Year)^REF^	Risk of Bias for					Overall Risk of Bias
	Selection	Performance	Detection	Attrition	Reporting	
Ko et al. (2022)^17^	Moderate	Low	Moderate	Low	Low	Moderate
Costa et al. (2022)^18^	Low	Low	Moderate	Low	Low	Low
Ahmed et al. (2021)^19^	Moderate	Moderate	Moderate	Low	Low	Moderate
Moussa (2020)^20^	Moderate	Low	Low	Low	Low	Low
Tan et al. (2020)^21^	High	High	Moderate	High	Moderate	High
Chuhuaicura et al. (2019)^22^	Low	Low	Moderate	Low	Low	Low
Fukutake et al. (2018)^23^	Low	Low	Low	Low	Low	Low
Banu et al. (2016)^24^	Low	Low	Moderate	Low	Low	Low
Matsuda et al. (2014)^25^	Low	Low	Low	Low	Low	Low
Luraschi et al. (2013)^26^	Low	Low	Low	Low	Low	Low
Ohkubo et al. (2013)^27^	Low	Low	Low	Low	Low	Low
Kimoto et al. (2011)^28^	Low	Low	Moderate	Low	Low	Low
Hosoi (2011)^29^	Low	Low	Low	Low	Low	Low

Risk of bias definitions: *Low*, the study design, methods, and analysis suggest minimal risk of bias in this category; *Moderate*, the study has some concerns or limitations in this category, but they do not significantly affect the overall conclusions; *High*, the study has major limitations that likely introduce substantial bias in the results.

### Ko et al. (2022)^17^—Masticatory Function and Cognitive Impairment

This population-based matched case-control study explored the relationship between changes in masticatory function and cognitive decline in older patients, specifically looking at longitudinal data from Korean individuals. Results indicated that in the dementia group, the total functional tooth unit (T-FTU, a measure of masticatory function) significantly decreased over time, while no such change was observed in the control group. Regression analysis revealed that neglected missing teeth and previous alcohol consumption were significant risk factors for dementia. Their findings suggest that neglecting prosthetic restoration could be causally linked to the onset of dementia.

### Costa et al. (2022)^18^—Dental Prostheses and Brain Activity

This systematic review aimed to establish the link between oral prosthetic rehabilitation and regional brain activity. Analysis of the eight included studies found evidence of increased regional blood flow and cerebral activity while using dental prostheses. The results suggest that prosthetic rehabilitation has a positive influence on brain function, preserving and potentially restoring neurological health.

### Ahmed et al. (2021)^19^—Dental Prostheses and Cognitive Function

This systematic review by Ahmed et al. found that dental prostheses play a significant role in preventing cognitive impairment. The studies collectively demonstrated that prostheses can enhance cognitive function in patients with dementia-related diseases and neurodegenerative conditions, acting as a protective factor against cognitive decline.

### Moussa et al. (2020)^20^—Brain Activity and Cognitive Function

This study examined the effects of restoring posterior occlusal contacts in controlled type 2 diabetic patients with removable partial dentures (RPDs). Assessments using electroencephalography (EEG) and Mini-Mental State Examination (MMSE) scores revealed significant improvement in brain function and cognitive status after one month of wearing RPDs. This suggests that restoring occlusal function with RPDs has a positive impact on brain activity and cognitive performance in diabetic patients.

### Tan et al. (2020)^21^—Rehabilitation and Cognitive Outcomes

This pilot study focused on the effects of progressive oral implant rehabilitation on masticatory performance, neurocognitive function, and oral health-related quality of life (OHRQoL). Four completely edentulous patients were observed, with improvements in masticatory performance and OHRQoL after transitioning from removable dentures to implant-retained prostheses. Neurocognitive changes were also observed, indicating a positive association between prosthetic adaptation and cognitive function.

### Chuhuaicura et al. (2019)^22^—Mastication and Cognitive Decline

This literature review analyzed studies on the relationship between mastication and cognitive decline. The findings suggest that masticatory function plays a protective role in patients with cognitive impairment and neurodegenerative diseases. Increased brain blood flow, particularly in regions like the frontotemporal cortex and caudate nucleus, was linked to chewing intensity, supporting the idea that mastication may help preserve cognitive function.

### Fukutake et al. (2018)^23^—Cognitive Function and Oral Perception

This study focused on how cognitive function affects oral perception in older adults living independently. The analysis showed a significant relationship between cognitive function, the number of teeth, and the use of removable dentures in determining stereognostic scores. Cognitive impairment, even in its preclinical stage, was associated with reduced oral perception, emphasizing the importance of maintaining cognitive health for better oral function.

### Banu et al. (2016)^24^—Brain Activity in Edentulous Patients

Banu et al. performed a pilot study comparing brain activity and cognitive function in edentulous patients using conventional dentures and mandibular overdentures supported by two implants. They found that implant-supported overdentures improved both brain activity (measured by EEG) and cognitive scores. This suggests that the functional improvement from implant-supported dentures contributes to better mental health and cognitive performance.

### Matsuda et al. (2014)^25^—Vertical Dimension and Electroencephalograms in Denture Users

This study investigated how changes in the vertical dimension of occlusion affected brain activity in complete denture users. The results showed no significant changes in neuronal activity before and after chewing, but a significant decrease in occlusal force was observed with a −3 mm vertical dimension of occlusion. This finding suggests that changes in occlusal force can influence both brain activity and psychological state.

### Luraschi et al. (2013)^26^—Neuroplasticity in Prosthodontics

This study used functional magnetic resonance imaging (fMRI) to examine brain activity changes in patients adapting to new complete dentures. The authors found that brain activity during motor tasks involving dental occlusion recovered to baseline levels after three months, suggesting that prosthodontic treatment adaptation leads to neuroplastic changes, improving both chewing efficiency and bite force.

### Ohkubo et al. (2013)^27^—Occlusion and Brain Function

Ohkubo et al. analyzed the effect of occlusion on brain function, focusing on the sensorimotor cortex. Results indicated that chewing and other movements in the oral area stimulate cerebral cortex activity, which could help prevent the degradation of brain function. Their study suggests that occlusal interference devices, splints, and implants all impact brain function through their effects on oral movements.

### Kimoto et al. (2011)^28^—Chewing and Brain Activity in Edentulous Patients with Overdentures

This preliminary report by Kimoto et al. investigated brain activity changes during gum chewing in edentulous patients who transitioned from complete dentures to implant-supported overdentures. The results showed a decrease in brain activity in the prefrontal cortex with implant overdentures, highlighting differences in neural responses between the two prosthodontic treatments.

### Hosoi et al. (2011)^29^—Dentures and Brain Function

Hosoi et al. examined brain function activity in elderly patients using complete or partial dentures. They found that denture use improved brain function activation, with occlusal contact area and force influencing the extent of activation. Their results suggest that restoring proper occlusal contact in denture users can enhance brain activity and function.

## DISCUSSION

The World Health Organization (WHO) classifies the aging population into different age categories, with middle-aged individuals being those aged 45–59 years, elderly or early old age spanning 60–74 years, old or late old age covering 75–89 years, and very old referring to those aged 90 years and above; this classification serves as a framework for understanding and addressing the needs of the aging population in public health and geriatric care.^30^

By 2050, low- and middle-income countries are projected to have one in five people aged over 60. India will experience a significant rise in its elderly population due to advancements in medical technologies, leading to increased life expectancy. By 2100, India will have one elderly person for every three working-age individuals.^31^

Nutrition plays a vital role in dental health, which, in turn, influences cognitive functioning.^32^ Chewing impacts cardiac activity, with evidence of increased sympathetic stimulation and elevated oxygen levels in the prefrontal cortex and hippocampus area. A study by Seraj et al. found that elderly individuals with good chewing efficiency showed improved cognitive capacity and memory retrieval compared to the beginning of the study.^33^ Additionally, both removable dental prostheses and implant-supported prostheses significantly improve nutritional status and oral health perception in the elderly, leading to increased cognitive capacity.^34^

The 13 studies presented herein had various outcomes that emphasized the significance of removable dental prostheses in influencing the cognitive capacity of individuals. The outcomes relate to five important areas that merit consideration and are discussed below.

### Impact of Natural Dentition versus Edentulism

Differences in brain activity were noted between patients with complete natural dentition and patients with partial and total edentulism.^23^

Neuro-cognitive tests have shown significant differences between the two groups, with individuals having a complete set of natural teeth displaying better cognitive function compared to those with missing teeth. Cognitive changes at a preclinical stage can alter oral perception, which may serve as an early indicator of neurological health in dental practice. However, it should be noted that assessment of cognitive function changes should not solely rely on the presence or absence of natural teeth or dentures; dental professionals need to consider this as a variable.^25^,^27^

Studies utilizing 3-T fMRI have revealed differences in brain activity in individuals who are missing teeth, specifically reduced blood oxygenation level-dependent contrast. The loss of dental receptors may result in decreased synapses between peripheral areas and the central nervous system, potentially impacting brain activity in edentulous individuals.^35^

This suggests a disconnection of stimuli, contributing to the observed differences between individuals with natural teeth and those who are partially or completely edentulous, particularly in neurocognitive tests, stereognostic capacity, and neuronal activity in the sensorimotor cortex. However, the specific impact of dental status on stereognostic capacity remains inconclusive and requires further investigation to avoid biased or generalized conclusions.^36^

These data indicate that the absence of dentition may pose a risk of declining brain activity, but using dentures could potentially improve this function.

### Edentulous Patients versus Denture Users

Differences in brain activity between edentulous patients and patients using removable dental prostheses were observed.

Edentulous patients generally suffer from notable impairments in brain activity, cognitive scores, and mental state when compared to individuals using removable dental prostheses. The latter group experiences enhanced brain function and cognitive scores, and this improvement is attributed to occlusal interference devices that have a positive effect on the sensorimotor cortex.^37^

To obtain unbiased results, further studies with more cases are necessary. Nevertheless, it is crucial to acknowledge that removable dental prostheses play a vital role in assisting patients to enhance their nervous system’s ability to adapt both structurally and functionally to various environments. Devices like occlusal interference devices, splints, and implant prostheses contribute significantly to these positive outcomes.^38^

The brain’s adaptability enables anatomical and functional regeneration, leading to the recovery or adaptation of lost functions and the creation of new signaling pathways. The prosthetic stimulus in the edentulous area activates synaptic activity, thereby promoting increased neuronal communication.^39^ Edentulous patients experience limitations in brain activity due to the absence of receptors in the periodontal ligament, which necessitates adaptations to establish communication with the central nervous system in response to oral stimuli.

The placement of prostheses, especially removable ones, serves as an alternative means to stimulate the brain activity of edentulous patients.^20^ These prostheses significantly improve brain function, cognitive scores, and functional, sensory, and motor capabilities, while also providing comfort, functionality, and aesthetic improvement for the patient.^24^,^29^

### Mastication and Its Cognitive Impact

The physiological process of chewing (mastication) plays a crucial role in brain activity, particularly from the perspective of older patients with removable dental prostheses.^22^,^28^ The results of this review suggest the presence of a sensory system that receives and processes stimuli, involving various receptors to facilitate the flow of information.^40^

Interestingly, the structures of the stomatognathic system are interconnected with input stimuli, generating information that is then processed by the neural mechanisms of masticatory patterns. This processing leads to responses in brain activity, indicating a close link between the stomatognathic and central nervous systems. The modulated response is subsequently fed back to the stomatognathic system where its structures play crucial roles in various human activities, impacting emotions, instincts, taste, hunger, food discrimination, saliva secretion, and swallowing.^41^

### Tooth Loss and Brain Activity Variations

This review demonstrated the importance of determining the relationship between tooth loss and variation in brain activity. Our findings demonstrated the positive effects of dental prostheses on motor activity, cognitive activation, and neurocognitive abilities, resulting in progressive improvement in evaluations. Lower cognitive function was significantly associated with poor oral perception, which is influenced by the number of teeth and the use of removable dental prostheses. This latter observation is also notably linked to stereognostic scores.^18^,^42^

A prospective analysis of patients with dentures and mandibular overdentures supported by two implants highlights their importance in improving the patient’s mental state and functionality. Furthermore, using adapted replacement dentures during motor tasks leads to changes in brain activity, indicating a relationship between the removable dental prostheses and brain function.^19^,^43^

Chewing stimulates brain activity in the cerebral cortex, potentially contributing to the prevention of cognitive decline. Differential activity is observed in the prefrontal cortex during chewing. Overall, brain function activity improves with the enhancement of complete and partial dentures, emphasizing the need for necessary adjustments to enhance patients’ quality of life.

The results of this review established correlations between diminished synapses connecting peripheral areas with the central nervous system due to the loss of dental receptors.^21^ These correlations are evident in variations of brain activity observed through various evaluation techniques, with tangible improvements seen when patients use prostheses.^44^

Consequently, tooth loss leads to reduced brain activity due to the lack of stimulus communication in the edentulous area. The provision of removable dental prostheses or implant-retained mandibular overdentures offers patients greater stability in brain function, leading to improved neurocognitive abilities and increased sensory information, among other benefits.

### Prostheses and Cerebral Changes in Edentulous Patients

This study revealed the importance of determining whether or not there is a relationship between removable dental prostheses use and physiological or adaptive cerebral changes in partially and completely edentulous patients.^17^

This study provided compelling evidence for the relationship between tooth loss and variations in cerebral activity, particularly affecting neurocognitive abilities and functional tasks, and masticatory performance, oral cavity, and cognitive function assessments.^26^ Removable dental prostheses play a crucial role in influencing neurocognitive changes and compensating for reduced sensorimotor function caused by tooth loss.

Use of EEG further supports the connection between removable dental prostheses and cerebral activity, with alterations in alpha waves indicating severe cognitive impairment in completely edentulous patients. Prosthetic rehabilitation in edentulous individuals leads to sequential improvements in brain activity and cognitive function.^45^

Significantly, the use of prostheses also improves motor tasks related to occlusion, transmitting sensory information to the brain, affecting psychological conditions, and influencing occlusal force when using complete dentures.^46^

This literature review also noted a link between cerebral activity and the mastication process, particularly with components of the stomatognathic system. Stimulation during mastication significantly influences cerebral function, contributing to enhanced mental health and regulating memory, learning, stimuli, and emotions.^47^

### Summary

Combined, the above findings indicate that cerebral activity undergoes alterations in edentulous patients, with modifications observed when removable dental prostheses provide stimuli from the environment, particularly in the cerebral cortex (voluntary movement control) and the motor cortex (related to the oral and maxillofacial region). Current research highlights the important and positive impact of removable dental prostheses on neurocognitive abilities and overall brain function in individuals with tooth loss.

## CONCLUSION

This study acknowledged that tooth loss, whether partial or total (edentulism), is a prevalent issue among elderly patients and has a notable impact on the oral cavity, which, in turn, can affect brain activity. The literature revealed differences in the observed brain activity between individuals with natural dentition and those who were edentulous, and these differences were correlated with neuro-cognitive test results and brain function.

The use of complete dentures was found to improve brain activity, indicating the importance of removable dental prostheses in addressing the effects of edentulism on cognitive function. However, the literature also revealed that edentulous patients with removable dental prostheses showed impaired brain activity and cognitive scores, suggesting that not all types of prostheses may have equally beneficial effects.

Tooth extraction and the use of prostheses were shown to have a positive impact on brain activity, functionality, and aesthetic appearance, ultimately enhancing the overall quality of life. Mastication, the process of chewing, was found to influence brain activity, emotions, and saliva secretion.

However, tooth loss without prostheses resulted in decreased brain activity due to reduced sensory stimulation from the oral cavity. On the other hand, removable dental prostheses were identified as effective in stimulating cerebral systems, supporting brain function, and promoting neurocognitive activities.

In conclusion, this study suggests that removable dental prostheses play a crucial role in positively influencing brain activity, mental health, oral health, and emotional well-being. This, in turn, can boost individuals’ confidence in personal, professional, and social aspects of life, highlighting the importance of addressing tooth loss in elderly patients through appropriate prosthetic solutions.

## Abbreviations

EEG: electroencephalography
fMRI: functional magnetic resonance imaging
MMSE: Mini-Mental State Examination
OHRQoL: oral health-related quality of life
PICO: Population, Intervention, Comparison, and Outcome
PRISMA: Preferred Reporting Items for Systematic Reviews and Meta-Analyses
RPDs: removable partial dentures
